# TNIP1 As A Potential Factor In Neuronal Survival, Growth and Differentiation in the Cerebellar Cortex

**DOI:** 10.1007/s12311-026-02080-5

**Published:** 2026-09-09

**Authors:** Carolina Göttsche Esperança Clara, Spoelgen Michael, Sebastian P. Roeser, Karl Schilling, Stephan L. Baader

**Affiliations:** 1 Institute of Anatomy, Anatomy and Cell Biology, University of Bonn, Nussallee 10, Bonn, D-53115 Germany; 2 Department of General and Interventional Cardiology/Angiology, Ruhr University Bochum, Heart and Diabetes Center NRW, Bad Oeynhausen, Germany

**Keywords:** Autophagy, Mitosis, Cerebellar Development, TNFAIP3-Interacting Protein, A20-Binding Inhibitor, ABIN1

## Abstract

Tumor Necrosis Factor-Induced Protein 1 (TNIP1) is a well-known ubiquitin-binding signaling molecule involved in innate and adaptive inflammatory processes. It has been implicated in diverse immunological diseases, but also associated with various neuro-psychiatric conditions, including Alzheimer’s disease, glioma formation, and demyelination. However, a systematic analysis of TNIP1 expression and function in the central nervous system is missing. Here, we combine immunohistochemical staining, Western blotting, quantitative PCR and the re-analysis of publicly available single cell- and in situ hybridization-mRNA datasets to document the expression of TNIP1 in the developing and adult murine cerebellum. We document that TNIP1 is broadly expressed in cerebellar neurons and macroglial cells, where it is localized to discrete puncta within cell nuclei and somata, including Purkinje cell dendrites. Both TNIP1 and its mRNA are developmentally regulated in the early postnatal cerebellum, and levels of the latter vary systematically across the cell cycle. Consistently, overexpression of TNIP1 in human embryonic kidney cells inhibits proliferation and activates cleaved caspase-3 expression. These observations support a role for TNIP1 in neur(on)al development and function and establish a paradigm to study the mechanistic basis of its function in the normal and diseased nervous system.

## Introduction

Tumor Necrosis Factor-Induced Protein 1 (TNIP1) is a polyubiquitin-binding protein involved in proteasomal protein degradation and linked to autophagy by its association with the autophagosomal marker protein LC3 [1–4]. TNIP1 has also been shown to activate the nuclear factor kappa B signaling pathway, thus promoting inflammatory processes at the transcriptional level [5, 6]. Changes in TNIP1 expression have been implicated in innate and/or adaptive immune responses and the genesis of inflammation-associate diseases such as systemic sclerosis, psoriasis, systemic lupus erythematosus or Sjögren’s syndrome [7–10].

More recently, genetic variations of TNIP1 have also been linked to pathologies of the central nervous system (CNS), including major depressive disorders, Alzheimer’s disease, fatal neurodegenerative disorder, demyelinating diseases, and glioma [11–16]. As TNIP1 is considered to maintain immune homeostasis [17], one may also speculate whether it modulates the phenotype of neurodevelopmental disorders, such as tuberous sclerosis complex, associated with neuroinflammation.

In contrast to malignant neural cells, the expression of TNIP1 in normal cells of the CNS has not been studied - or published - in any detail. Gene expression profiling has shown that TNIP1 is expressed within the CNS [18]. However, to our knowledge, the cells expressing TNIP1 in the CNS and possible regulators of its expression remain so far elusive.

As a first step towards elucidating the expression of TNIP1 in the CNS and to establish a basis for understanding its (mechanistic) involvement in CNS pathologies mentioned above, we investigated TNIP1 expression in the developing murine cerebellar cortex. This choice is based on the well-known structure of the cerebellum, its interconnectivity and details of physiological cellular processes taking place at distinct developmental time points (see [19] for a recent compendium on cerebellar developmental and clinical implications). The cerebellum is also an extensively documented paradigm for the study of fundamental processes underpinning brain development, from cell proliferation and specification to migration and synaptic integration [19]. Using immunohistochemical staining of mouse tissue and primary cell cultures, we show that TNIP1 is expressed in all cell types of the cerebellar cortex. TNIP1 signals appear as punctate staining within the nucleus, the cytoplasm but also in cellular processes. Overexpression of TNIP1 in human embryonic kidney cells affected proliferation and cell death. The current study not only demonstrates the distribution of TNIP1 expression in the cerebellum and in particular cell types, but also strongly supports its role in post-mitotic development of neurons and glia.

## Materials and Methods

### Mouse Handling

All mice used in this study were offsprings of C57BL/6J mice (Janvier, Paris, France) bread in our local animal facility approved by the local authorities. Overall, mice of either sex were used. While genetic variants of *TNIP1* have been associated with several conditions known to show a sex bias (e.g., [20]) no sex-related effects of Tnip1-deficency or Tnip1 expression have been reported in mice [21]. Consistently, interrogation of single cell expression data obtained from adult murine cerebella [22] (4 males, 2 females) do not suggest sex-related differences of Tnip1 expression (p_adj_ = 0.997 for *Tnip1*-RNA levels in Purkinje cells (PC); p_adj_ = 0.999 for *Tnip1*-RNA levels in all cells; bulk analysis using DESeq2). Therefore, in this baseline study, we focused on potential developmental effects without accounting for sex.

Mice had access to food and water ad libitum and were held at a constant light/dark rhythm of 12/12 hours between 20 and 24 °C and 40 to 70% humidity. The day of birth was designated as postnatal day 0 (P0). The birth of new pups was checked once a day. The following ages were used: P0, P3, P5, P9, P15 and adult (*P* > 59 days). Perfusion fixation was approved by the local authorities (the Landesamt für Natur, Umwelt und Verbraucherschutz/LANUV NRW Germany; permission number 84-02.2017.A120).

### Quantitative PCR

Quantitative reverse transcription PCR was done as described in [23]. Total RNA was prepared from newborn, 3-, 5-, and 7-day-old mouse cerebella using TrizolRNA kit (Invitrogen, Darmstadt, Germany, 15596018). It was then reverse transcribed using iScript according to the manufacturer’s protocol (BioRad, Munich, Germany, 1708841) and amplified using the DyNAmo Flash Probe qPCR kit (Thermo Fisher, Darmstadt, Germany, F455L) as per the supplier’s instructions. PCR was started with denaturation at 95 °C for 10 min followed by 40 cycles at 95 °C (15 s), and 60 °C (60 s). Afterwards the melting curve was determined in between 65 °C and 95 °C. Primers for TNIP1 (Mm01288484) and the reference gene Rn18s (Mm03928990) were obtained from Thermo Fisher. Quantitative analysis was performed using CFX Manager software (BioRad, Munich, Germany, 1845000). TNIP1 data were normalized to the reference gene to allow dependable and sensitive inter-run calibration and comparisons [24]. The cerebella of individual mice of a litter were processed independently, thus allowing us to use one litter to obtain one timeline. Three independent litters were used. Since the variability of qPCR data was high at P0 and rather small at P7, we used 5 cerebella from three litters for P3 and only 2 samples of two litters at P7. Using mice of one litter at different time points allowed us to use a linear mixed model to calculate changes over time. Since the overall effect of a single continuous variable (age) is addressed, multiple comparisons are not warranted. Prior testing for homogeneity of variance was done by Levene’s test.

### Immunohistochemical Staining of Tissue Section

Mice were perfused according to standard procedures as described before [25]. Briefly, mice were anesthetized with ketamine/xylacine (80/8 mg/kg i.p.) just before perfusion. Blood was replaced by Ringer solution (Fresenius, Bad Homburg, Germany), followed by 4% paraformaldehyde (PFA) and 0,2% glutaraldehyde (GA) buffered in phosphate buffered saline (PBS, pH 7.4). Brains were dissected and post-fixed in the same fixative for 1 h for newborn pups and up to 6 h for adult brains. Afterwards, brains were stored in PBS containing 0.1% sodium azide at 4 °C until cutting. 50 μm thick sagittal sections were prepared using a Leica VT1000S vibratome (Leica, Wetzlar, Germany). For antigen demasking sections were heated to 80 °C in 11.4 mM sodium citrate buffer (pH 6.0) for 30 min. Blocking of unspecific binding sites was done in 0.2% gelatin/2% goat serum diluted in PBS (pH 7.4) for 2 h. 1% Tween20 was added to ensure sufficient antibody permeation. Then, sections were incubated at 4 °C in the primary antibody solution diluted in the above blocking solution. The following antibodies and dilutions were used: anti-TNIP1 (Proteintech, Planegg-Martinsried, Germany, 15104-1-AP, rabbit, 1:100), anti-Calbindin D28k (Sigma-Aldrich, Taufkirchen, Germany, C9848, mouse, 1:1000), anti-GFAP (Merck-Millipore, Darmstadt, Germany, IF03L, mouse, 1:100), and anti-MBP (Chemicon, Limburg, Germany, MAB381, mouse, 1:500). After several washings, the secondary antibodies diluted 1:1000 in the same blocking solution were applied for 1 h (Alexa Fluor 488 goat-anti-rabbit and Alexa Fluor 546 goat-anti-mouse, Invitrogen, Darmstadt, Germany, A-11008 and A-11030, 1:500). Cell nuclei were stained in 1 µg/ml Hoechst 33258/PBS for 10 min. Sections were embedded in fluoromount G (Thermo Fisher, Darmstadt, Germany, 00–4959-52) and imaged using a Nikon laser scanning microscope A1 HD254/ECLIPSE Ti2-E) equipped with a 20x and 60x objective (Nikon, Düsseldorf, Germany). A pinhole size of 1 airy unit was chosen. To ensure comparability, the same gain and offset settings were used for ages to be compared. Pictures shown in the figures were obtained by combining five z-planes taken within the nuclear region of the depicted cell using maximum projections. All immunohistochemical stainings included negative controls for which the primary antibodies were omitted. These controls yielded no signal. All staining was done on at least two sections of three mice of different litters. All images were taken from the anterior lobe.

### Analysis of Single Cell RNA Data

Single cell RNA data for the adult and developing cerebellum generated and originally described by Kozareva et al. [22] and Vladoiu et al. [26] were obtained from the cellxgene repository (https://cellxgene.cziscience.com/collections/f70ebd97-b3bc-44fe-849d-c18e08fe773d) and the National Center for Biotechnology Information – Gene Expression Omnibus (NCBI GEO) repository (GSE118068), respectively. All analyses were implemented in R [27] using, inter alia, the packages Seurat, sctransform, glmGamPoi, sceasy, reticulate, scCustomize and DESeq2.

Analysis of adult cerebellar cells was based on the clustering and cell-type annotation established by Kozareva et al. [22]. Expression matrices for the developing cerebellum (embryonic day 14 - postnatal day 7) were combined in a single Seurat object. We selected for cells expressing > 500 and < 4800 transcripts and < 5% mitochondrial genes. Data were then normalized using sct-transformation [28] and integrated using Harmony [29]. Cell types were annotated based on canonical markers for the developing murine cerebellum [22, 30–32]. Cell cycle assignment was done using the ccAFv2 package [33].

### Primary Cerebellar Cell Cultures and Immunocytochemical Staining of Cultured Neural Cells

Dissociated cerebellar cell cultures were prepared as described [34]. Briefly, brains of newborn mice were dissected in ice-cold Hanks buffer containing 140 mM sodium chloride, 5.2 mM potassium chloride, 4.2 mM sodium hydrogen carbonate, 0.9 mM hydrogen phosphate, 3.3 mM calcium chloride and magnesium chloride each and 5.5 mM glucose. After mechanical and chemical dissociation in Trypsin-ethylenediaminetetraacidic acid (EDTA, GIBCO/Thermo Fisher, Darmstadt, Germany), cells were filtered and resuspended in neurobasal medium (GIBCO) containing B27-supplement (GIBCO), GlutaMAX (GIBCO) and penicillin/streptomycin (GIBCO) as suggested by the manufacturer. Cells were cultivated on glass cover slips coated with 0.02 mg/ml poly-L-lysine at 37 °C and 95% air/5% CO_2_ for 7 days, changing half of the medium every other day. Cells were fixed in 4% PFA/0.2% GA for 5 min at 37 °C and then kept in PBS until staining.

For immunocytochemical staining, cells were blocked in 10% fetal calf serum containing 0.45% Triton X-100 at room temperature for 1 h. They were then incubated with primary antibodies diluted in blocking solution at 4 °C overnight. Secondary antibodies were prepared in the same blocking solution and added for 1 h at room temperature. The following antibodies were used at the dilutions indicated: anti-TNIP1 (Proteintech 15104-1-AP, rabbit, 1:100), anti-TUBB3 (Sigma-Aldrich T8578, mouse, 1:100), anti-MAP2 (Sigma-Aldrich M4403, mouse, 1:100), anti-FABP7 (Abcam, Cambridge, UK, ab277622, mouse, 1:100), anti-OLIG2 (Millipore MABN50, mouse, 1:100) and the Alexa488- and Alexa 546-secondary antibodies (Invitrogen, goat anti-mouse and anti-rabbit, 1:800). Imaging was done using a Nikon confocal laser scanning microscope A1 HD25/ECLIPSE Ti2-E. To ensure comparability, identical settings for gain and offset were used throughout. Again, all analyses included negative controls for which the primary antibodies were omitted. These controls yielded no signal. Cultures were repeated once with comparable results.

### Human Embryonic Kidney 293 Cell Transfection

Human embryonic kidney 293 (HEK) cells were cultured in DMEM containing 1% Penicillin/Streptomycin, 10% FCS and 50 mM GlutaMax (GIBCO) at 37 °C in 95%air/5% CO_2_. HEK cells were transfected using the DharmaFECT Duo kit following the protocol of the manufacturer (Dharmacon, Rodgau, Germany, T-210-03). For overexpressing TNIP1 in HEK cells, the human TNIP1 sequence was amplified from pUNO1-hTNIP1a (InvivoGen, Toulouse, France), adding BamHI sites to both ends. The TNIP1 fragment was inserted into the pMini vector using a NEB PCR Cloning Kit (NEB, Frankfurt, Germany, #E1202S) as described by the manufacturer. Using BamHI restriction digests, the Tnip1 sequence was then ligated to a pcDNA3.1zeo vector [35]. The inserted sequence, the insertion site and the orientation were checked by sequencing. As a control, the enhanced green fluorescent protein sequence was inserted into the pcDNA3.1zeo vector the same way.

### Western Blotting

TNIP1 protein expression in HEK cells was monitored by Western blotting as described [36]. To do so, HEK cells were resuspended in 1 mL ice-cold 1 x PBS, centrifuged, and the cell pellet was lysed in 400 µL protein extraction buffer containing 50 mM TRIS pH 8.0, 150 mM NaCl, 1% Triton X-100, and Pefabloc SC-protease-inhibitor (Roth, Karlsruhe, Germany, A154). Samples were ultrasonicated, centrifuged at 13,000 x g for 10 min, and the protein concentration of the supernatant was determined using the BCA assay (Thermo Fisher, A55865). A commercially available Western blot gel (Criterion TGX precast gels, Bio-Rad) was used for separating proteins. Proteins were then blotted onto a polyvinylidene fluoride membrane using the Trans-Blot Turbo Transfer System of Biorad. After washing in Tris buffered saline and blocking in 5% powdered non-fat dry milk dissolved in tris-buffered saline (TBS), membranes were incubated with primary antibodies (TNIP1, rabbit polyclonal, Proteintech 15104-1-AP, 1:1000 and Actin beta, mouse monoclonal, Thermo Fisher 1:5000, AB_2537667) diluted in 5% powdered milk TBS/0,5% Tween20 at 4 °C overnight. The following day, membranes were washed and secondary antibodies were applied (Anti-Rabbit-IgG, horseradish peroxidase (HRP)-coupled, AB_2313567, 1:30.000, Jackson ImmunoResearch, Hamburg, Germany, and Anti-Mouse-IgG-HRP coupled, AB_10015289, 1:10.000, Jackson ImmunoResearch). The membranes were then incubated with Westar Supernova enhanced chemoluminescent solution (Cyanagen, Bologna, Italy) and the resultant chemiluminescence was captured using an iBright CL 1500 imaging system (Thermo Fisher, Darmstadt, Germany). Images were densitometrically quantified using the integrated density measure of ImageJ [37]. To calculate relative expression levels, the intensity of the TNIP1 band was related to the Actin beta band intensity. Western blot data were repeated once with comparable results. Differences in expression were calculated using the Wilcoxon rank sum test implemented in R.

Western blotting of cerebellar tissue was done as described above. Up to three cerebella were pooled from one litter and used at each time point. In total, two litters were analyzed and statistically evaluated by restricted maximum likelihood estimates in linear mixed-effects models using the lmer function implemented in R’s lme4 package. Since the aim of this analysis was identifying changes of a single continuous variable (age), multiple comparisons are not indicated.

### Density-Dependent Gene Expression in HEK Cell

Data for comparing *Tnip1*-mRNA expression in HEK cells plated at low and high densities were obtained from the GEO database GSE249290 [38]; *n* = 3 independent assays per condition). *Tnip1-*mRNA levels were quantified relative to that of *Actin beta*. Significant differences in the means were calculated using ANOVA testing implemented by the “aov” function in R.

### Immunocytochemistry with HEK 293 Cells

Immunocytochemical staining of HEK cells was done as described above for dissociated cerebellar cultures. The following antibodies and dilutions were used: anti-TNIP1 (Proteintech 15104-1-AP, rabbit, 1:1000), anti-cleaved Caspase-3 (Merck/Millipore AB3623, mouse, 1:500), anti-phospho-histone H3 (pHH3, Invitrogen PA5-17869, rabbit, 1: 500). Secondary antibodies were anti-mouse IgG Alexa 488 (Invitrogen, A-11003, 1:800), anti-rabbit IgG Alexa 488 (Invitrogen A-11008, 1:800), anti-rabbit IgG Alexa 546 (Invitrogen A11035, 1:800).

While double staining for TNIP1 and cleaved Caspase-3 was done simultaneously, double staining for pHH3 and TNIP1 had to be done sequentially as both primary antibodies were raised in rabbits. The strict localization of the pHH3 signal to the nuclei of a subset of TNIP1 positive cells documents that sequential staining was specific for the respective antibodies. Subsequently, cells were counterstained with Phalloidin and Hoechst 33,258. Phalloidin- tetramethylrhodamine-isothiocyanate (TRITC) was used at 50 µg/mL in PBS for 40 min, and Hoechst 33,258 at 1 µg/ml in PBS for 10 min.

### Morphometry and Statistics for HEK Cell Analysis

Images were taken using an A1 HD25/ECLIPSE Ti2-E Nikon laser scanning microscope equipped with a 60X oil immersion objective (CFI Plan Apochromat Lambda D 60X Oil). Pixel scan size was set at 2048 pixels. Images were taken with a zoom factor of 1.0 and 4.0, (see scale bars shown). The image acquisition settings for the photomultiplier tube, high voltage, offset and laser of each color channel were kept constant to minimize technical bias towards control (GFP-transfected) or TNIP1-transfected cells. For morphometry, regions were selected in which single cells could be unambiguously discriminated. Four to six images were taken per condition and culture. All measurements included three independent cultures. This way 15 views were evaluated for pHH3 expression analyses and cell size measurements, and 9 images were taken for counting cells.

To analyze TNIP1 staining, integrated intensities of TNIP1 grayscale values were obtained using ImageJ and related to the number of cells present in the visual field analyzed. Cells were counted in high magnification views. Caspase-3 staining intensities were analyzed analogously. To analyze pHH3 expression, pHH3-positive cells were counted and related to the total number of cells within a visual field. Soma sizes of HEK cells clearly expressing TNIP1 were measured after delineating the borders of these cells with the freehand selection tool in ImageJ. For all morphometrical analyses performed, an inter-rater test was applied to calculate the single-random-raters inter-rater correlation coefficient (R package psych, function ICC, model 2), which was above 0.95 for all assays. All data were depicted in histograms and were non-parametrically distributed. Integrated staining intensities for TNIP1, Caspase-3 and cell areas, as well as counts of pHH3-positive cells were compared using Wilcoxon rank sum tests. No multiple comparison was used since data were compared along a single continuous variable (age). All statistical analyses were done in R.

## Results

### Expression of TNIP1 During Cerebellar Development

**Fig. 1:**
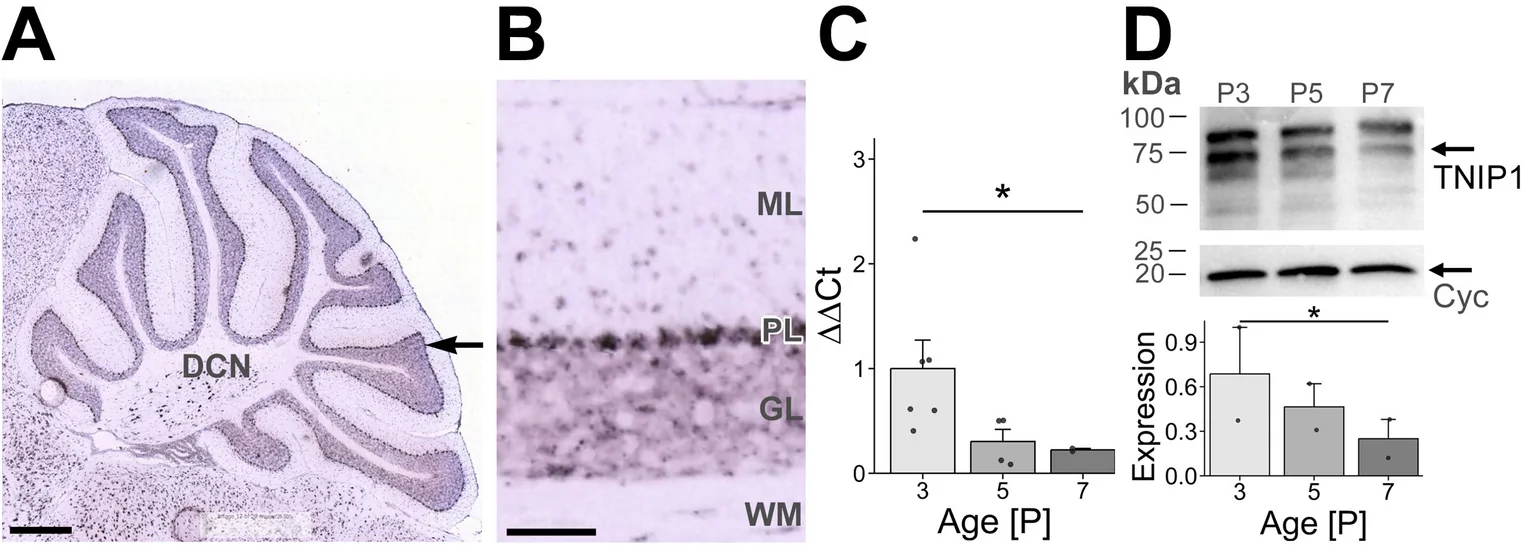
(**A**, **B**) In situ hybridizations reveals *Tnip1*-mRNA expression within the cerebellar cortex, in particular the Purkinje cell layer (arrow), and the deep cerebellar nuclei (DCN). (**B**) Higher magnification allows recognition of *Tnip1*-mRNA in the Purkinje cell layer (PL), the granule cell layer (GL), the molecular layer (ML) and the white matter (WM). Images are taken from the Allen Brain Atlas. Bar = 1 mm for A, and 100 μm for B. (**C**) Quantitative PCR analysis of *Tnip1*-mRNA expression during early postnatal cerebellar development revealed that expression decreased between P3 and P7 (n_P3_ = 5, n_P5_ = 4, n_P7_ = 2; *p* = 0.045; linear mixed-effect model). (**D**) Western blotting of cerebellar protein extracts again suggests that levels of TNIP1 decrease during early cerebellar development (*n* = 2; *p* = 0.032; linear mixed-effects model). The immunoreactive band at ~ 80 kDa is due to post-translationally modified TNIP1 (see main text and discussion). Asterisks in C and D mark a significant decrease over age (*p* < 0.05)

To get a first impression of TNIP1 expression in the murine brain, and in particular the cerebellum, we referred to the in situ hybridization data available in the Allen Brain Library (https://mouse.brain-map.org/experiment/show/68443063) [39]. As documented there, *Tnip1*-mRNA is expressed throughout the adult brain. In the cerebellum, expression is particularly prominent in the Purkinje cell layer of the cerebellar cortex (Fig. 1A, B) and in large cells of the cerebellar nuclei. Higher magnifications of the cerebellar cortical layers demonstrate that besides expression in the Purkinje cell (PC) layer, a fainter *Tnip1*-mRNA signal is also present over the granule cell (GC) layer, where a few larger cells stand out due to their intense staining. The molecular layer contains dispersed *Tnip1*-positive cell somata most likely representing molecular layer interneurons. A few stained cells observed within the white matter represent either oligodendrocytes or astrocytes (Fig. 1B).

To assess expression of *Tnip1*-mRNA during early postnatal development of the cerebellum, we first performed quantitative PCR analysis of cerebella obtained from newborn, three-day-old, five-day-old and seven-day-old mice (Fig. 1C). These time points were chosen to cover the period of transient PC loss due to apoptosis and/or autophagy around postnatal day (P) 3 [40, 41] and prominent proliferation within the external granule cell layer at P7. Between P3 and P7, *Tnip1*-mRNA expression decreased about 3.3-fold. Afterwards, expression levels remained constant until adulthood.

Next, we analyzed TNIP1 expression at the protein level. Western blotting of protein extracts obtained from P3, P5 and P7 old cerebella revealed two prominent TNIP1-positive bands at about 70 and 80 kDa. TNIP1 has an apparent molecular weight of 72 kDa. The additional band at around 80 kDa which can frequently be seen in datasheets of antibody suppliers and in publications [1, 42, 43]. Consistent with mRNA data, TNIP1 protein expression dropped significantly between P3 and P7.

The distribution of *Tnip1*-mRNA as revealed by in situ hybridization with adult sections clearly suggests that *Tnip1*-mRNA is expressed in several different cell types. We used comparative immunohistochemical staining to detail TNIP1-protein expression at the cellular level. In order to verify that the TNIP1 antibody used here specifically stains TNIP1, we used several approaches. Knockout of TNIP1 causes prenatal lethality due to various organ failures [1] which prevented the use of sections of knockout animals as a negative control. We therefore used sections stained with secondary antibodies only and cultured cells genetically engineered to overexpress TNIP1 (see below) as controls.

**Fig. 2:**
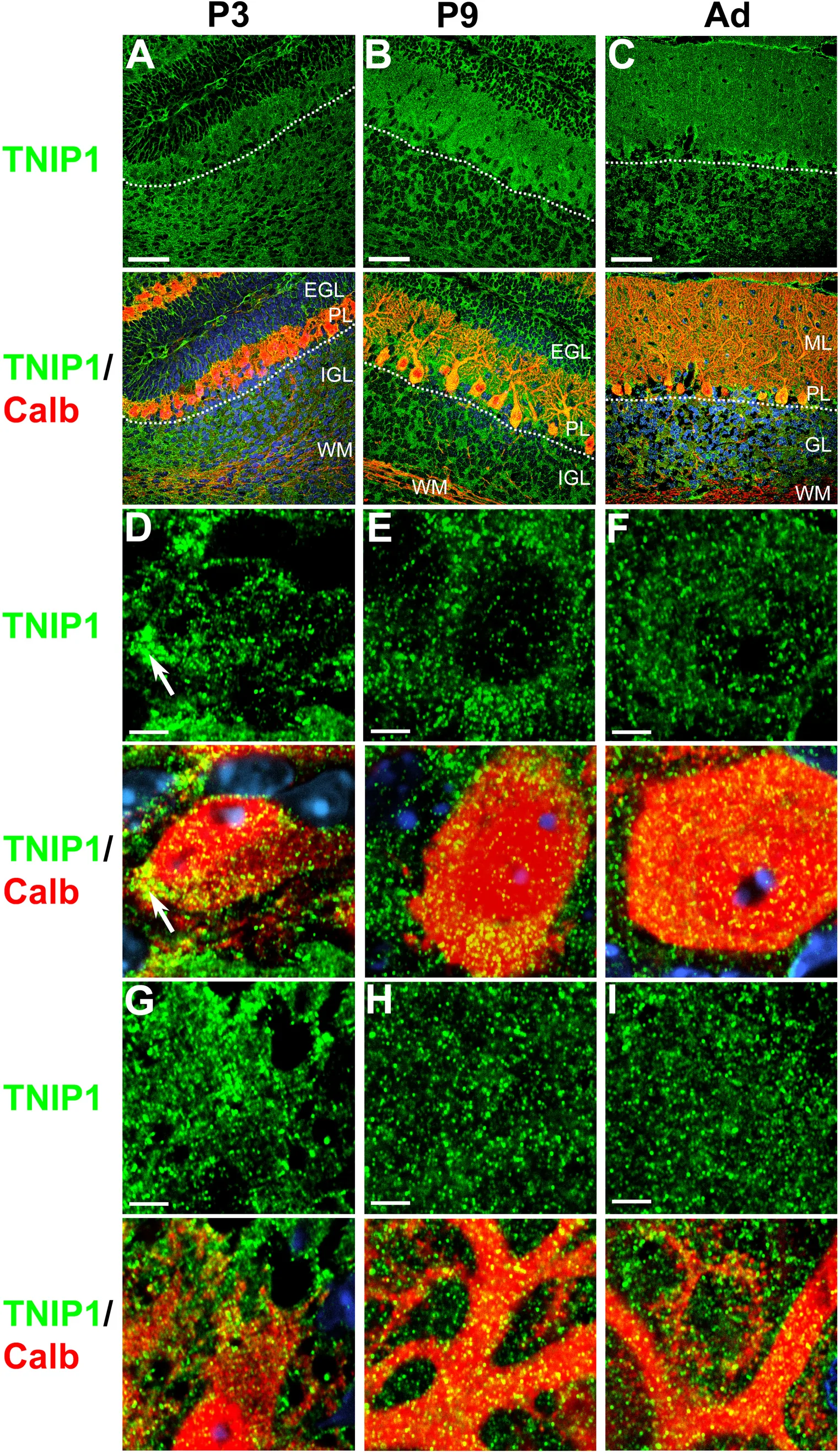
TNIP1-protein is expressed in cerebellar Purkinje cells throughout postnatal development. Cerebellar vibratome sections derived from three-day-old (P3), nine-day-old (P9) and adult mice (Ad) were stained with antibodies against TNIP1 (green) and Calbindin-D28K (Calb, red). TNIP1 appears in granular structures of the cytoplasm, within the nucleus and in processes throughout development. In sections of P3 (D) animals, TNIP1-positive puncta are particularly dense close to the axon hillock (arrow). Bar is 50 μm in A-C and 5 μm in D-I. For all images, single TNIP1 stainings and double stainings (with Calb) are shown. EGL external granule cell layer, ML molecular layer, PL Purkinje cell layer, IGL inner granule cell layer, WM white matter. Representative images are shown from slices made from three different cerebella at each age

**Fig. 3:**
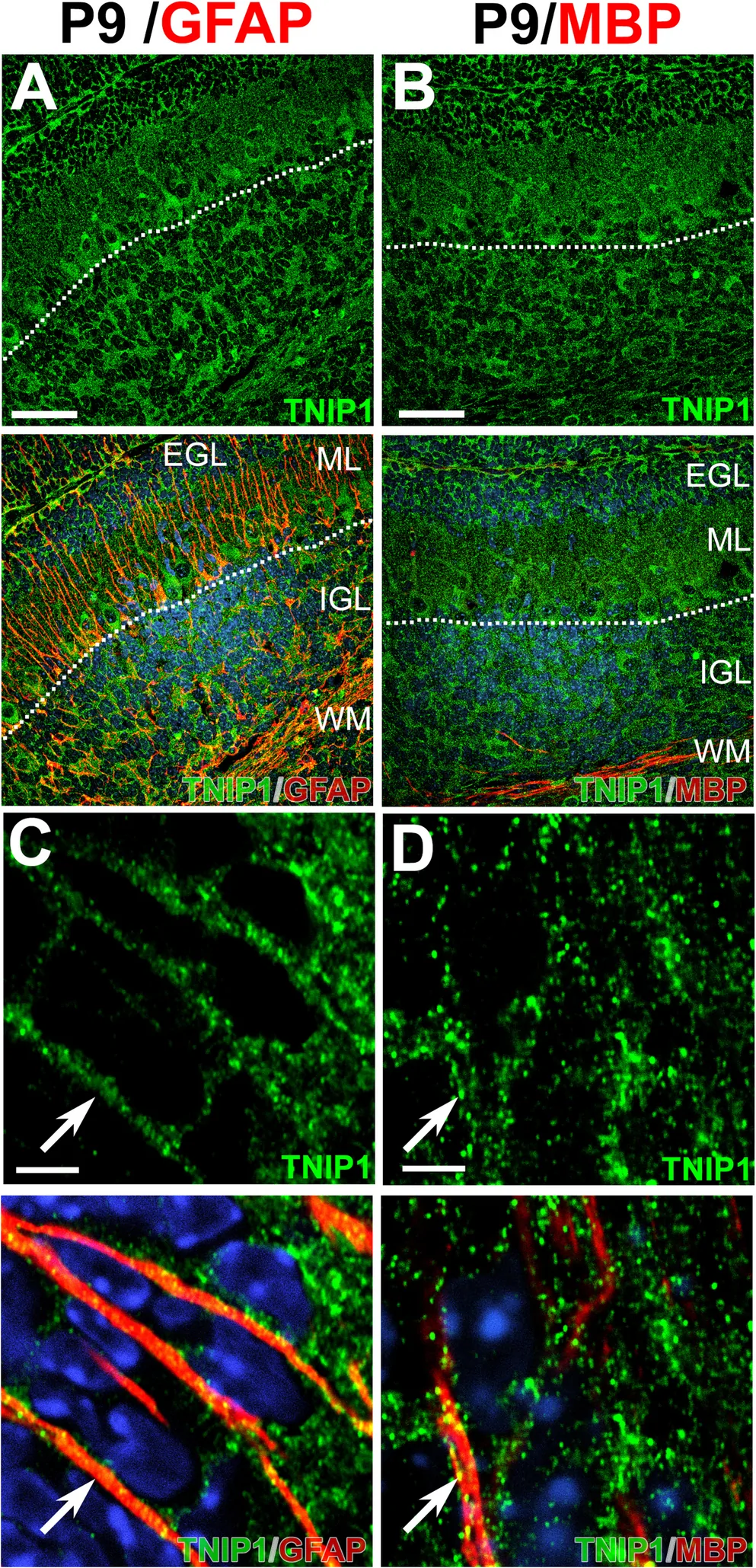
TNIP1 is expressed in GFAP-positive astrocytes and in the white matter where it colocalizes with MBP-positive oligodendrocytic processes. Cerebellar vibratome sections derived from nine-day-old (P9) mice were immunohistochemically stained with antibodies against TNIP1 (green) and glial fibrillary acidic protein or myelin basic protein (GFAP or MBP, both in red). TNIP1 appears in granular structures of processes at P9 which most probable represent Bergman glia fibers (arrows in C) and myelinated axons (in D). Bar is 50 μm in A-B and 5 μm in C-D. For each time point and antibody staining, a TNIP1 only staining is shown in the top row and the corresponding double staining below. EGL external granule cell layer, ML molecular layer, IGL inner granule cell layer, WM white matter. Representative images are shown from slices made from three different cerebella

Immunohistochemical staining of cerebellar sections derived from 3-day-old, 9-day-old and adult mice highlight a punctate pattern within the cytoplasm and the nucleus of PCs (Fig. 2). The large size and clearly defined axons and dendrites of these cells also enabled us to compare TNIP1 distribution in these distinct neuronal compartments. While uniformly distributed in the nucleus and dendrites of PCs, axon hillocks seem to contain a higher number of these puncta (arrows in Fig. 2D). This concentration was clearly visible in cells at P3, was much less prominent in P9 and no longer visible in adult cells. The increase in mRNA expression observed by qPCR was not paralleled by an increased immunosignal in single PCs. As may be taken from Fig. 2, immunoreactivity for TNIP1 is not restricted to PCs. Double staining for the glial markers GFAP and MBP showed that TNIP1 is also expressed by astrocytes and oligodendrocytes (Fig. 3). In particular, TNIP1 immunoreactivity is clearly associated with the straight, radially oriented GFAP-positive processes of Bergmann glia present in the molecular layer of the cerebellar cortex (Fig. 3C). In addition, TNIP1 is also found in scattered astrocytes within the granule cell layer (Fig. 3A). Common to all cell types is its appearance as punctate structures. Lastly, the TNIP1 staining pattern seen over the developing and adult molecular and granule cell layers (Fig. 2) suggest that TNIP1 might also be expressed by granule cells and inhibitory interneurons. However, the small size of these cells and the complexity of the neuropil does not allow verifying whether these signals originate from these neurons or the surrounding glia.

**Fig. 4:**
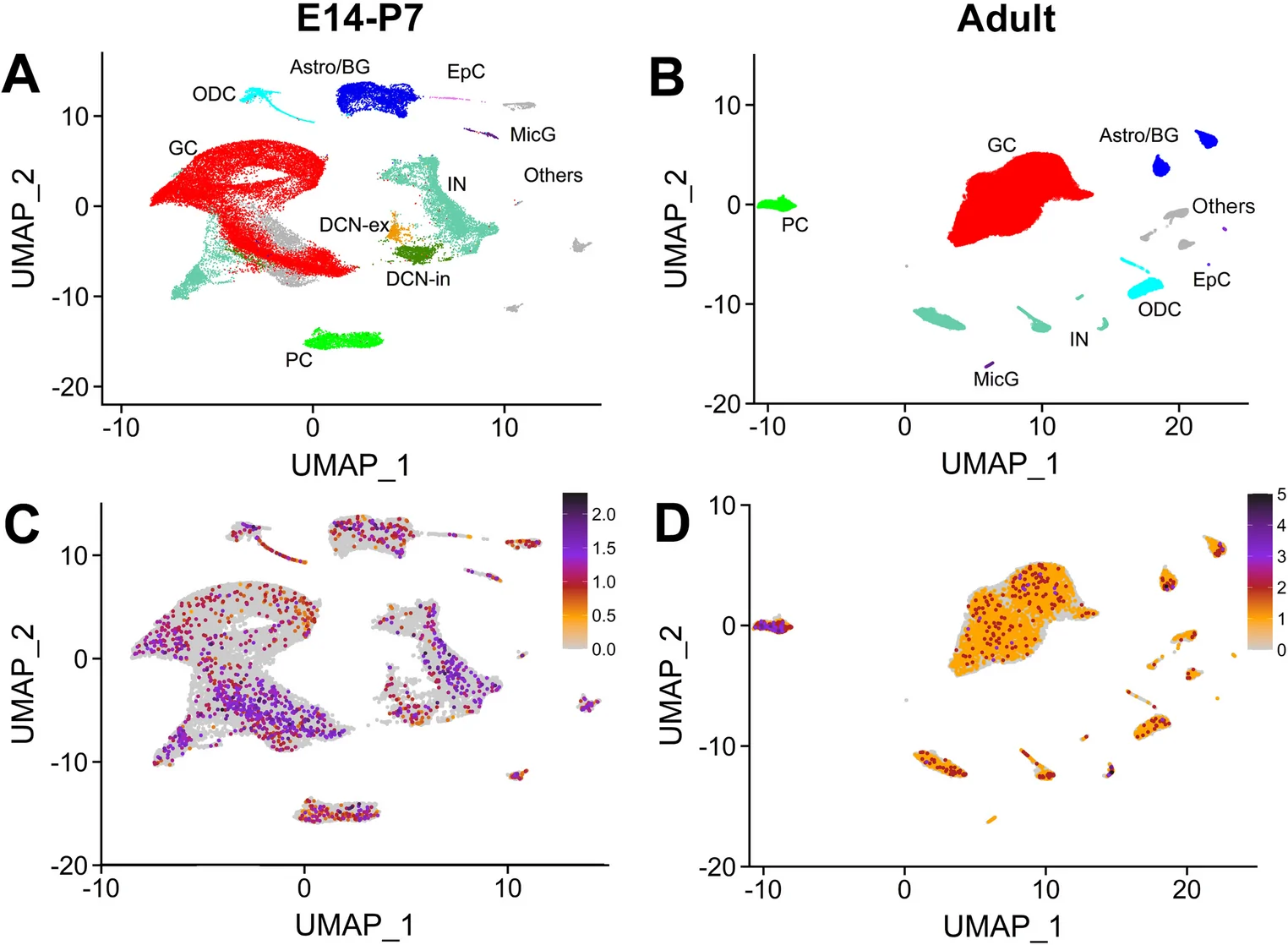
*Tnip1*-mRNA expression in cells of the developing (**A**, **C**) and adult (**B**, **D**) cerebellum. Panels A and B indicate cell types, and C and D document *Tnip1*-positive cells. Note the stronger expression of *Tnip1*-mRNA in adult Purkinje cells. Astro/BG astrocytes/Bergmann glia, DCN-ex excitatory neurons of the deep cerebellar nuclei, DCN-in inhibitory neurons of the deep cerebellar nuclei, EpC ependymal cells, GC granule cells, IN inhibitory interneurons, MicG microglia, ODC oligodendroglial precursor cells, PC Purkinje cells

To further ascertain the rather widespread expression of TNIP1 indicated by the above results, we analyzed *Tnip1*-mRNA expression using two publicly available single cell RNA datasets [22, 26] obtained from adult and developing cerebella. As documented in Fig. 4, *Tnip1*-mRNA may be found in all major cell types present in the adult cerebellar cortex. Other than neurons, Bergman glia, astro- and oligodendroglial cells, cells of non-neuronal lineages, like microglia, meninges and vascular cells are also *Tnip1*-positive (designated summarily as “Others” in Fig. 4). The set of developing cerebellar cells analyzed also comprises deep nuclear cells; these also expressed *Tnip1*.

### Expression and localization of TNIP1 in cultured cerebellar neurons

**Fig. 5:**
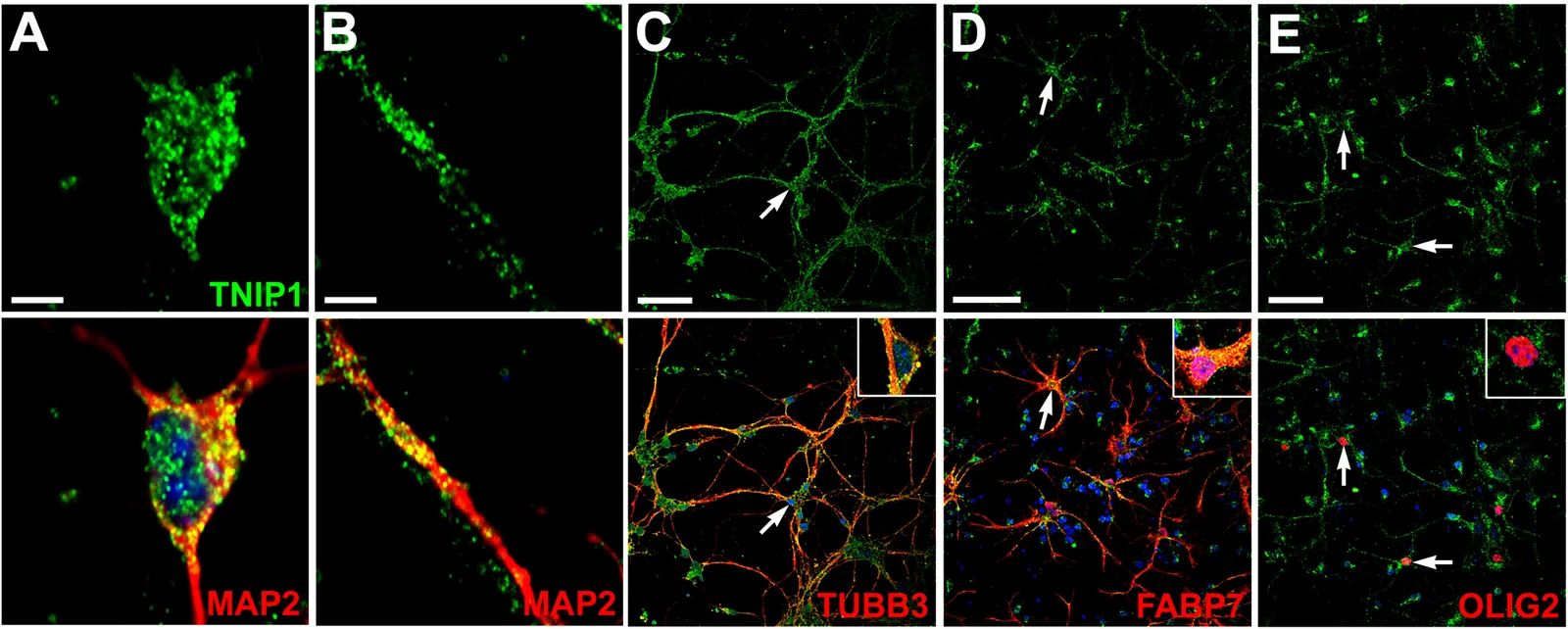
TNIP1 expression in dissociated cerebellar cultures prepared from newborn mice. Cultures were stained with antibodies against TNIP1 (green, applies to A-E), and co-immunostained (in red) for either MAP2 (A and B), tubulin beta 3 (TUBB3, C), brain lipid binding protein (FABP7, D) or oligodendrocyte transcription factor 2 (OLIG2, E). TNIP1 appears in a granular pattern within the cytoplasm, the nucleus and in cellular processes of all cell types (see also inlays in C-E). Arrows in C-E mark individual examples of stained cells. Neurons depicted in figures A and B are most likely larger interneurons such as basket or stellate cells as their somata are much larger than that of granule cells. Bars are 5 μm in A and B, 50 μm in C-E. Representative images are shown from cultures made from three different cerebella

Immunocytochemical staining of dissociated cerebellar cultures prepared from newborn mouse pups and grown in vitro for 10 days revealed that all major neural cell types expressed TNIP1 also under these reductionistic conditions. Thus, TNIP1-immmunoreactive puncta colocalized with the neuronal markers MAP2 and tubulin beta 3 (TUBB3), the astroglial marker FABP7 (also known as BLBP), and also with the oligodendrocyte transcription factor 2 (OLIG2), expressed in oligodendroglial precursor cells. As on sections from intact cerebella, TNIP1 staining reveals a granular staining pattern within the nucleus, the cytoplasm and also in processes (Fig. 5).

**Fig. 6:**
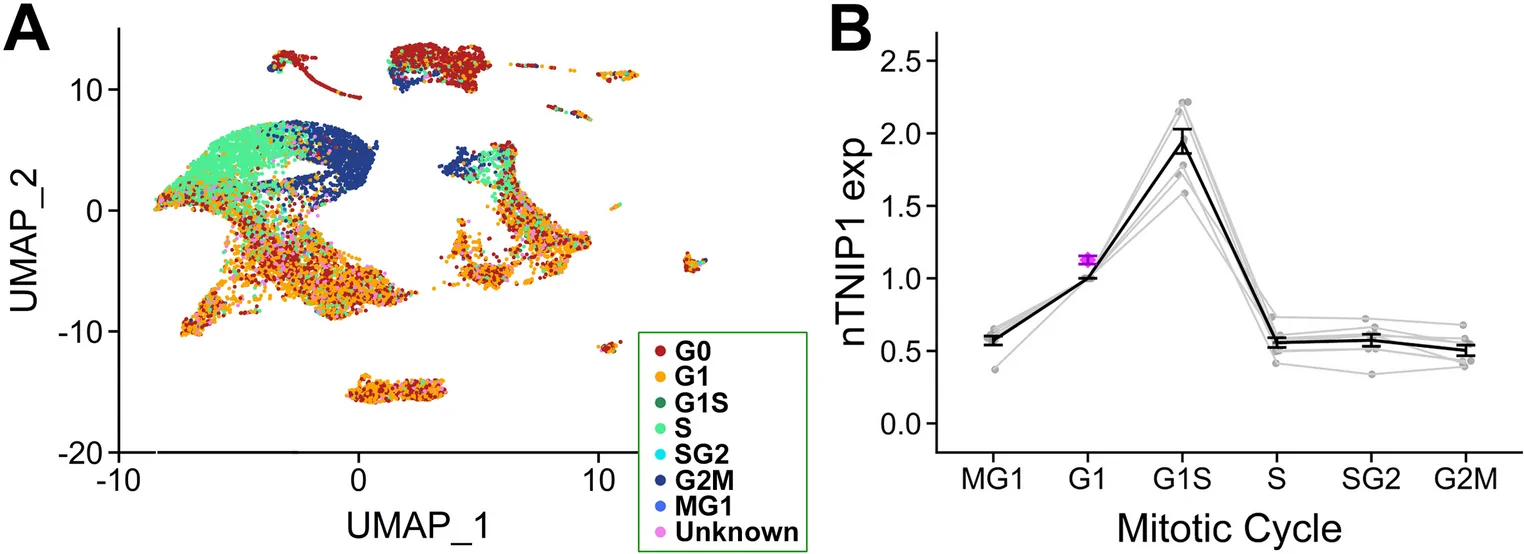
*Tnip1*-mRNA expression during cell cycle phases. (**A**) Cell cycle phases of cells from the developing cerebellum are indicated. (**B**) *Tnip1-*mRNA expression in developing cerebellar cells relative to eight established reference genes (Actb, Atp5f1, Ccnc, Gapdh, Pgk1, Tmem234, Uqcrfs1 and Ywhaz shown in light gray). To allow comparative presentation of the resulting ratios, these were scaled such that values at G1 equaled unity (1). The black line depicts the mean of reference lines, error bars give SEMs. The value for G0 is plotted along with that of G1 (magenta color). Note that a stronger expression of *Tnip1*-mRNA could be observed in clusters comprising preferentially G0/G1 cells. MG1 early G1 phase, G1S late G1 phase, SG2 early G2 phase, G2M late G2 phase

The above results indicate that TNIP1 is broadly expressed in cerebellar cells and, importantly, that its expression changes dynamically as cerebellar cells are generated and start to differentiate. Therefore, we asked whether *Tnip1-*mRNA expression varies across the cell cycle of nascent cerebellar cells. A comparison of Fig. 4A with Fig. 6A suggests, that cells in G1/G0 express higher levels of *Tnip1-*mRNA than cells in other phases of the cell cycle. To assess this quantitatively, we again took advantage of the publicly available single cell data of Vladoiu et al. [26]. Cells obtained from embryonic day 12 (E12) to 7-day-old animals were grouped based on their position in cell cycle using the ccAVv2 procedure, and the bulk expression in these groups was then normalized by the expression of several “housekeeping” genes established as references for quantitative PCR of murine material. These included Actb, Atp5f1, Ccnc, Gapdh, Pgk1, Tmem234, Uqcrfs1 and Ywhaz. As shown in Fig. 6B, *Tnip1-*mRNA levels increased during G1, then dropped to about half of the level seen in G1 as cells transit into S-phase, and stayed at this lower level until cells re-entered G1. Expression of *Tnip1-*mRNA in G0 was comparable to that in G1.

### Overexpression of TNIP1 in HEK cells causes cell death and diminishes proliferation

**Fig. 7:**
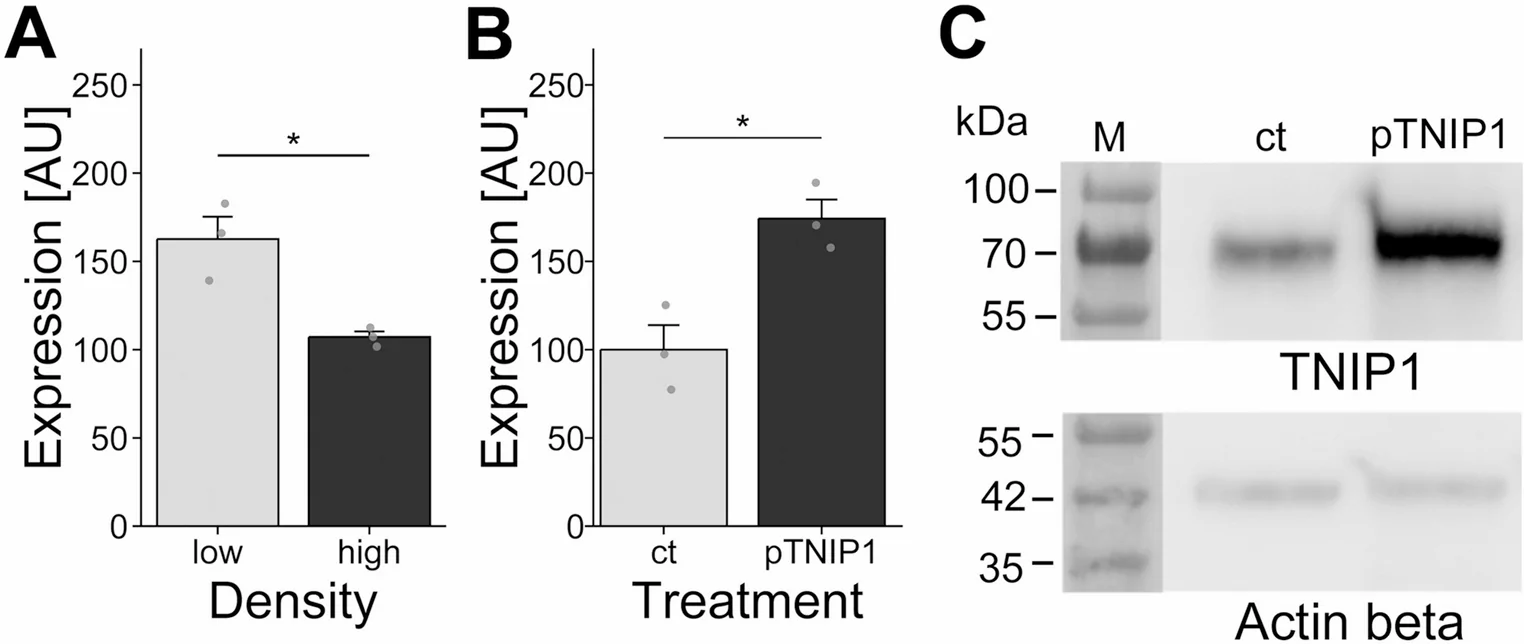
TNIP1 expression in HEK cells. (**A**) Cell density affects expression of *Tnip1-*mRNA in cultured HEK cells. Four independent analyses were published and are shown (*n* = 4; *p* = 0.013; ANOVA model). (**B**, **C**) Forced overexpression of TNIP1 in HEK cells substantially increased expression of TNIP1 protein (pTNIP1) (*n* = 3; *p* = 0.040; Wilcoxon Rank sum test). Control cells (ct) were transfected with GFP and grown to the same density as TNIP1-transfected cells. Actin beta was used to document equal loading of gels. Asterisks in A and B mark significant differences at a p level below 0.05

To further probe the interdependence of TNIP1 expression and cell proliferation, we overexpressed *Tnip1* in human embryonic kidney 293 cells (HEK cells). This cell line was chosen as it is easy to handle and readily amenable to genetic manipulation and analysis. We first assessed the effects of cell density on *Tnip1*-mRNA expression in HEK cells by interrogating the data associated with the publication of [38]. This revealed that sub-confluent HEK cells expressed substantially higher levels of *Tnip1*-mRNA than confluent cells (Fig. 7A). Based on this observation, we made sure that all subsequent comparisons between control and TNIP1-overexpressing cells were based on analyses of cultures at the same level of confluence. Cell density was monitored visually following nuclear staining and by assessing *actin beta* expression. We also based comparisons on TNIP1-protein levels.

**Fig. 8:**
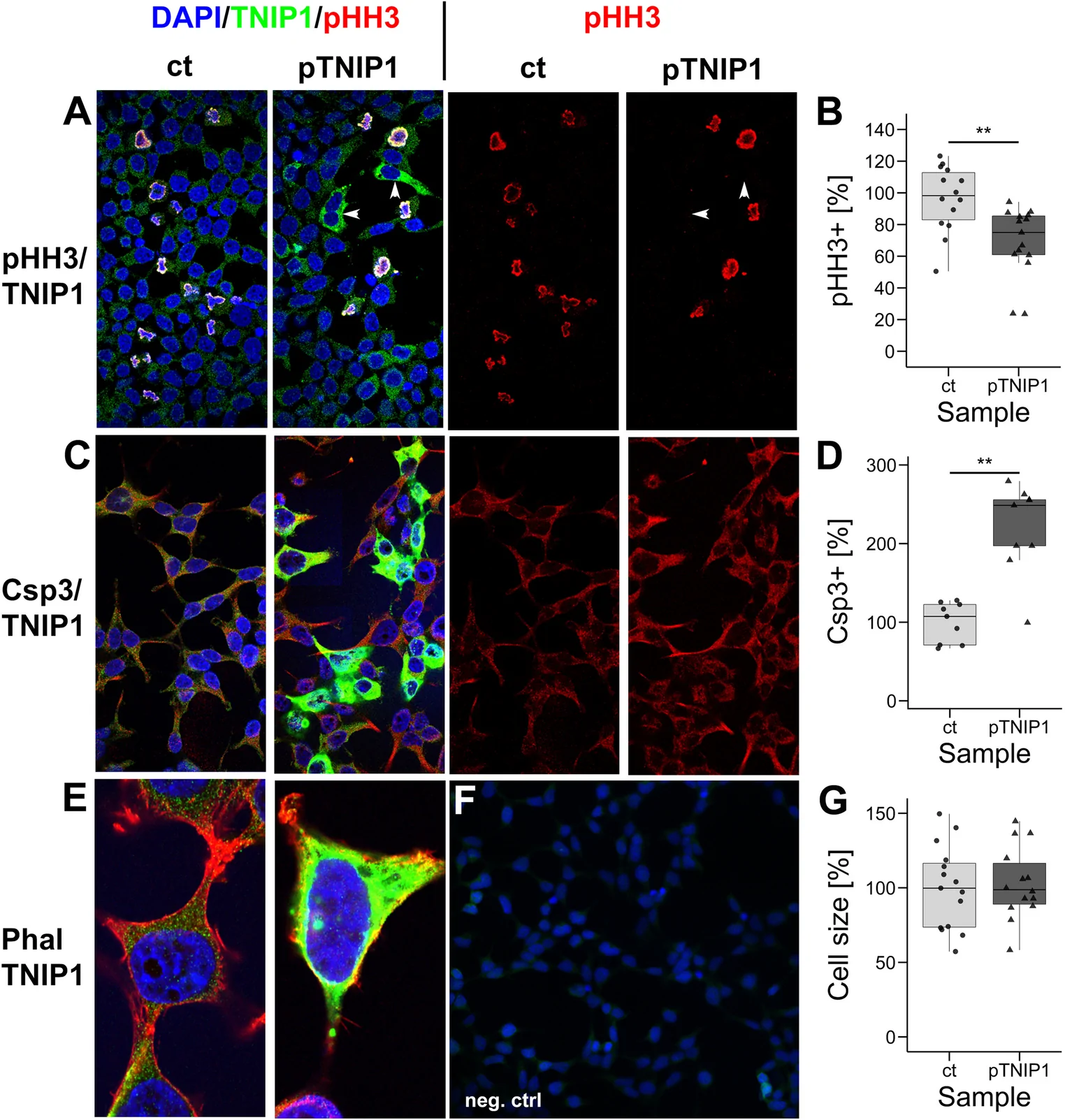
TNIP overexpression in HEK cells decreased phosphorylated histone expression (pHH3; panels A and B; *n* = 15; *p* = 0.0028) and increased cleaved caspase-3 expression (Csp3; panels C and D; *n* = 9; *p* = 0.0020), but had no effect on cell size (E and G; *n* = 15). Panel F shows a negative antibody control. Statistics of quantitative measures are based on three independent biological replicates (Wilcoxon Rank sum test, ** *p* < 0.01)

Forty-eight hours after transfection, HEK cells transfected with a plasmid from which TNIP1 is expressed under control of a CMV promoter expressed significantly more TNIP1 protein than control cells transfected with an EGFP containing vector (Fig. 7B-C). Increased TNIP1 expression was also clearly visible following immunocytochemical staining (Fig. 8). Immunocytochemical staining also revealed that transfection induced overexpression of TNIP1 was, as expected, variable between individual cells (Fig. 8). Increased TNIP1 expression was clearly visible in all cells, and exceeded base-line levels about twofold in some 35% of the cells (Fig. 7A, C, E, columns 1 and 2).

When cultures were double stained for TNIP1 and pHH3 which consistently stains chromatin at the metaphase of mitosis [44], significantly less pHH3-positive cells could be detected in TNIP1 overexpressing cells than in control cells (Fig. 7B). In contrast, TNIP1 overexpressing cells expressed significantly higher levels of cleaved Caspase-3 (Fig. 7D), the activated form of Caspase-3 associated with apoptotic cell death [45].

Lastly, we asked whether TNIP1-overexpression affects HEK cell morphology, taking cell size as a quantifiable proxy. As summarized in Fig. 8G, cell size was not affected by levels of TNIP1 expression. Also, upon visual inspection, the overall shape and appearance of cells was not appreciably changed upon forced TNIP1 overexpression. It should be mentioned that cultures stained with the secondary antibody only (negative controls) did not reveal any signal above background (Fig. 8F).

We thus can conclude that TNIP1 overexpression enhances apoptosis and diminishes proliferation, but has no detectable effect on cell size of cultured HEK cells.

## Discussion

The present findings document that TNIP1, best known for its involvement in the regulation of immune responses, is broadly expressed in neural cells of the adult and developing cerebellum. Expression levels change dynamically during the early postnatal phase of cerebellar development and across the cell cycle of proliferating neural cells. TNIP1 protein was found to be localized to fine puncta the subcellular distribution of which was also subject to developmental changes.

The broad expression of TNIP1 immunoreactivity in developing and adult cerebellar neurons and glia, as well as the doublet of bands seen in immunoblots for TNIP1 may warrant some consideration from a technical perspective. First, it should be reiterated that the signals obtained in immunohistochemical staining and western blotting were strictly dependent on the inclusion of the first antibody. The specificity of the latter is underscored by the discrete punctuate staining observed, which is consistent with the known spatial association of TNIP1 in defined organelles or vesicular structures (e.g., [46–48], as has been documented also by an antibody-independent approach using TNIP1-EGFP fusion constructs [49]. Further, antibody staining and mRNA expression are concordant, to the point that in adult tissues, TNIP1-immunoreactivity appears particularly prominent in Purkinje cells, as does expression of its mRNA (compare Figs. 1A, and 2). As shown by Shinkawa et al. [46] and also by Yin et al. [50], the two TNIP1-immunoreactive bands on immunoblots, best visible when gels are not heavily loaded, are caused by differential posttranslational modification, notably phosphorylation and possibly ubiquitination ([47], Figs. 5A and 6A). In conclusion, the antibody used allows reliable analysis of TNIP1 expression.

While expression of *Tnip1*-mRNA is well documented in the Allen Brain Atlas [39], data on the expression of its protein product in the adult and developing brain has, to the best of our knowledge, not been published. TNIP1 has extensively studied and well documented function(s) in the immune system, where it was found to limit exuberant inflammatory responses by regulating (macro-) autophagy (e.g. [4, 47]). Autophagy is also a key mechanism for the proper development of the nervous system and essential to maintain its functionality in the adult [51–53]. The findings summarized here suggest a regulatory role of TNIP1 in this, similar to that in the immune system, which may affect multiple aspects of neurogenesis, from the regulation of stem cell proliferation to axogenesis (for reviews, see [52, 54].

The cell cycle-related variation of the *Tnip1*-mRNA, which codes for an autophagy-regulated protein, is an interesting observation of this study. The fluctuation seen in cells of the perinatal cerebellum, the consistent variation of its expression in sub-confluent and confluent HEK293 cells, and the reduced numbers of mitotic cells seen in cultures of TNIP1-overexpressing HEK293 cells all suggest that TNIP1 may impinge on cell proliferation. This is also supported by the enhanced proliferation observed in clear cell renal carcinoma cells following antisense-mediated down-regulation of TNIP1 [55]. Of note, activity and subcellular localization of TBK1, which phosphorylates TNIP1 and thus targets it towards degradation, is also cell cycle-related [56, 57]. Given the rapid degradation of TNIP1 following phosphorylation by TBK1 [46, 47] implies that not only *Tnip1-*mRNA, but also its protein varies across the cell cycle. The continuous presence of two TNIP1-immunoreactive bands in protein extracts of the developing cerebellum (Fig. 1D) indicate that TNIP1 is post-translationally modified as cerebellar cells proliferate extensively and differentiate. Consistently, in non-stimulated HEK cells, a rather homogenous cell population, only a single band was observed, consistent with the data presented by Shinkawa et al. [46] and Yin et al. [50], who detailed a stimulus-dependent phosphorylation of TNIP1. The presence or absence of the double band might therefore indicate the distinct functional or differentiation states of cells. The regulation of the cell cycle by an autophagy-related protein may at first glance sound surprising, but both processes are closely connected by multiple signaling pathways and have implications in tumor growth, senescence and cytokinesis [58].

How might TNIP1 affect, or be affected, by the cell cycle? A parsimonious, if possibly simplistic idea posits that levels of TNIP1 compete with mitosis-regulating genes activated by TKB1-mediated phosphorylation (including PLK1, CEP170, NuMA, Cdc20, and Cdh1a; cf. [59]). These genes are all broadly expressed in the perinatal cerebellum, as may be taken, e.g., from the scRNA data of Vladoiu et al. [26]. In addition to its anti-proliferative effects, forced overexpression of TNIP1 uncoupled from the cell cycle also induced apoptosis in HEK293 cells. This begs the question whether it may also be involved in apoptotic cell death as it occurs in the developing cerebellum [60].

The documentation of TNIP1 expression in the cerebellum and its developmentally dynamic regulation during normal development suggest the cerebellar system as a starting point for elucidating the various neurological conditions that have been associated with TNIP1. So far, these include amyotrophic lateral sclerosis [61, 62], Alzheimer’s disease [63, 64] and/or hippocampal sclerosis [65], frontotemporal lobar degeneration with neuronal inclusions [15], glioma [12, 66], and major depressive disorders [14]. While the cerebellum may not be directly involved in all of these conditions, its histological setup is well known, basic physiological functions are described and it is readily accessible to experimental and genetic manipulation in vivo (see e.g. [67, 68], to mention just two seminal publications). This knowledge makes the cerebellum a promising paradigm to unravel the function of TNIP1 in the CNS under normal conditions and following selected challenges. Our data also document that TNIP1 expression is maintained under reductionistic conditions in primary cultures of the perinatal cerebellar anlage. In these, cellular communication and intracellular pathways may be efficiently targeted by pharmacological means not applicable in vivo. Primary cerebellar cultures are also readily accessible to functional, genetic and morphological analysis (e.g., [34, 69]).

Arguably, an intriguing question here will be how TNIP1 expression, posttranslational modification, and functional turnover are mechanistically regulated in neurons and glia. Data obtained with cells of the immune system pinpoint the central role of TBK1 in the regulation of TNIP1 [46, 47, 50]. While TBK1, the only identified phosphokinase acting on TNIP1, is broadly expressed in (healthy) neural cells (see, e.g., the data associated with the publications of Vladoiu et al. [26] and Kozareva et al. [22]; see also the Allen Brain Atlas), upstream regulators of TBK1 in immune cells (e.g., Toll-like receptors, or T-cell receptors) are not, at least not under physiological conditions, expressed by cerebellar neurons, and only weakly in subsets of cerebellar macroglia. Another intriguing issue is whether and how TNIP1 might interact with synaptic function, given that key molecules involved in its turnover and sequestration (GABA type A receptor-associated protein GABARAP and its paralogs GABARAPL1 and GABARAPL2 [4, 47] also bind to the GABA_A_ receptor subunit Gamma 2 and thus enhance their intracellular transport and surface expression [70, 71]).

## Conclusion

The widespread expression of TNIP1 in the CNS, as detailed here for the cerebellum, brings up the question whether the potential association of TNIP1 with neurological conditions as mentioned above is due to its function in neural cells, or in immune cells interacting with neurons and macroglia (see e.g. [72, 73], or both. The widespread expression of TNIP1 in the CNS pinpoints its implication in development leading to prenatal lethality upon gene disruption [1, 74] and supports the efforts put into targeting TNIP1-associated pathways for the treatment of peripheral autoimmune diseases.

## Data Availability

Single cell RNA data for the adult and developing cerebellum were obtained from the cellxgene repositotry (https://cellxgene.cziscience.com/collections/f70ebd97-b3bc-44fe-849d-c18e08fe773d) and the NCBI GEO repository (GSE118068). Data for comparing Tnip1-mRNA expression in HEK cells plated at low and high densities were obtained from the GEO database GSE249290.
